# Bilateral corneal perforation due to MRSA keratitis in a crosslinking patient

**DOI:** 10.3205/oc000072

**Published:** 2017-08-15

**Authors:** Zackery Oakey, Kevin Thai, Sumit Garg

**Affiliations:** 1 Gavin Herbert Eye Institute, University of California, Irvine, USA; 2 Touro University California, Vallejo, USA

**Keywords:** corneal collagen crosslinking, keratoconus, visual acuity

## Abstract

**Introduction:** The cornea may become infected and perforated after epithelium-on collagen crosslinking.

**Case presentation:** A healthy 33-year-old male who underwent corneal collagen crosslinking in both eyes developed a purulent keratitis and bilateral corneal perforations, requiring bilateral penetrating keratoplasties. He was exposed to methicillin resistant staphylococcus aureus (MRSA) by a family member with a tracheostomy and was treated with MRSA-directed antibiotics. After prolonged recovery and treatment of his infection, he had acceptable but limited uncorrected visual acuity, with excellent corrected visual acuity.

**Conclusion:** While epithelium-on crosslinking is commonly thought to be associated with a lower risk of postoperative infection, this case illustrates that even epithelium-on treatment may present the patient with a risk of infection. Patients in higher risk groups who are exposed to infectious disease may be more predisposed.

## Introduction

Keratoconus is a non-inflammatory ectasia that can be treated with corneal collagen crosslinking (CXL). Due to its molecular weight, the active crosslinking agent riboflavin does not readily penetrate to the deeper layers of the cornea and therefore an epithelium-off technique, as described in the Dresden protocol, is most commonly used to allow for increased riboflavin diffusion [1]. Keratitis caused by herpes virus, acanthamoeba, staphylococcus aureus, or even MRSA has been described after CXL [2]. The majority of infectious cases have been after epithelium-off crosslinking. Here we report a case of bilateral corneal perforations after epithelium-on collagen crosslinking for keratoconus when a bandage contact lens was used following the procedure. 

## Case description

An otherwise healthy 33-year-old African American male with an 11-year history of progressive keratoconus underwent bilateral epithelium-on collagen crosslinking at an outside clinic. His pretreatment best corrected visual acuity was 20/30^-1^ OD and 20/30 OS. 

Per review of the records, crosslinking was performed in both eyes using an epithelium-on protocol. Riboflavin was instilled every 5 minutes, as was proparacaine every 10 minutes, for 90 minutes until saturation of the corneal stroma was noted using a hand light. Fractionation was performed at 15 seconds on and 15 seconds off of UV light at a 3.0 inch distance with an energy of 3.0 mW/cm^2^ (measured by an energy meter). There were no reported complications. The corneal epithelium was intact after the procedure. A bandage contact lens (BCTL) was placed over both eyes and the patient was prescribed moxifloxacin 0.5% QID OU for 1 week, bromfenac 0.07% QID OU for 1 week, and prednisolone acetate 1% QID OU over 4 weeks that would be tapered slowly. 

On post-treatment day 4, the patient returned to the outside clinic complaining of severe pain OS starting the night before. There were new superficial corneal opacities, a hypopyon, and possible corneal perforation OS. A culture was performed, the BCTL was removed, bromfenac and prednisolone were discontinued OS and the patient was transferred to the Gavin Herbert Eye Institute (GHEI) for further care.

When the patient arrived at GHEI his vision was 20/70 OD and hand motion (HM) OS. The left eye exhibited diffuse conjunctival injection, a diffuse corneal infiltrate with gross ectasia and perforation along with a diffuse hypopyon. The right eye appeared to be unaffected. An emergent penetrating keratoplasty (PKP) and anterior chamber washout was recommended. The transplantation and washout were performed without complication and the patient was started on prednisolone acetate 1% and moxifloxacin 0.5% QID OS and ordered to continue prednisolone acetate 1% QID OD and moxifloxacin 0.5% QID OD per the referring doctor.

On postoperative day one from the PKP OS, the patient complained about new pain, irritation, and decreased visual acuity OD as well. He was using all recommended medications as described above. His visual acuity was HM OD and 20/80 OS. The right eye conjunctiva was injected and its cornea had a new 5 mm central infiltrate with an equally sized epithelial defect, an apical bulge and new purulent discharge. All of these findings were felt to be consistent with impending perforation. The left was healing appropriately. There was no evidence of recurrent infection OS and his IOP measured 21 mmHg by Tonopen. Given that the patient had a new epithelial defect, infiltrate and impending corneal perforation, we recommended a penetrating keratoplasty OD. 

On postoperative day one PKP OD, postoperative day two PKP OS, cultures returned from the first PKP OS showing many colonies of pan-resistant methicillin resistant Staphylococcus aureus (MRSA). The patient was recommended to undergo directed topical fortified vancomycin every 2 hours, topical trimethoprim/polymyxin B ointment every 2 hours OU, topical prednisolone acetate 1% QID OU, aggressive lubrication with nonpreserved artificial tears along with oral doxycycline and ascorbate. 

Given the severity of the patient’s course and no clear source for the MRSA, we recommended that the patient and his family be tested for MRSA, noting that his daughter had a tracheostomy. The patient later reported that he and his daughter wer positive for MRSA. He was given a course of trimethoprim/sulfamethoxazol (TMP/SMX) by his primary care physician.

By post-PKP month 3, his CDVA was 20/20 OU with a rigid gas permeable CTL. Specular microscopy showed healthy appearing endothelial cells bilaterally. 

## Discussion

Corneal collagen crosslinking is generally a safe treatment that rarely leads to secondary infection. Nevertheless, infections can occur and be visually devastating. Common organisms including Herpes virus, Pseudomonas aeruginosa, Staphylococcus aureus, Staphylococcus epidermis, and Acanthomoeba have been implicated and are generally reported after patients undergo the epithelium-off technique. Many times patients have concurrent systemic diseases such as atopy and diabetes mellitus [2], [3].

Given the clinical history of exposure from his daughter’s tracheostomy, the patient’s and family’s culture results, and good response to directed therapy, we feel that the most likely etiology of the patient’s corneal perforations was MRSA. The patient was colonized with MRSA and may have likely contracted from his daughter’s tracheostomy. 

What may have facilitated penetration through intact corneal epithelium is not yet clear. Although CXL was performed with the epithelium intact and was noted to be present after the procedure, the patient developed bilateral epithelial defects after the procedure. Whereas classically Neisseria, Corynebacterium, Haemophilus aegyptius, and Listeria bacteria are among those known organisms to have the potential to disrupt epithelial tight junctions and thereby lead to infection promulgation, MRSA is not usually listed among them. Some have theorized that the cross-linking process itself or the placement of a contact lens after the procedure may lead to microscopic trauma to allow for organisms not typically associated with infections over an intact epithelium [4]. Use of a bandage contact lens, while not always required following epithelium-on crosslinking, is often used to maximize patient comfort and may be related to the development of infection in this patient with intact epithelium.

Unlike other cases of MRSA keratitis after CXL, our patient did not have a history of any atopic disease although its incidence is 35% in patients with kerataconus [5], [6]. These factors can account for a higher incidence of MRSA seen in patients with Keratoconus treated with CXL. 

## Conclusions

Although CXL is relatively safe, rare complications can occur. While the epithelium-on technique is felt to be more protective to infectious complications, our case may present an exception. Appropriate preoperative planning including review of systemic medical history and physical examination are generally needed to avoid poor outcomes even when a reportedly less risk-laden method is used. Our case seems to imply that information pertaining to the patient’s exposure to infectious pathogens can be used to manage postoperative infections and/or prevent them. 

## Notes

### Competing interests

The authors declare that they have no competing interests.

### Funding

Supported in part by the “Research to Prevent Blindness” grant. 
